# Maltodextrin as a Drying Adjuvant in the Lyophilization of Tropical Red Fruit Blend

**DOI:** 10.3390/molecules28186596

**Published:** 2023-09-13

**Authors:** Yaroslávia Ferreira Paiva, Rossana Maria Feitosa de Figueirêdo, Alexandre José de Melo Queiroz, Francislaine Suelia dos Santos, Lumara Tatiely Santos Amadeu, Antônio Gilson Barbosa de Lima, Thalis Leandro Bezerra de Lima, Wilton Pereira da Silva, Henrique Valentim Moura, Eugênia Telis de Vilela Silva, Caciana Cavalcanti Costa, Plúvia Oliveira Galdino, Josivanda Palmeira Gomes, Douglas Alexandre Saraiva Leão

**Affiliations:** 1 Science and Technology Center, Federal University of Campina Grande, Campina Grande 58429-900, Brazil; antonio.gilson@ufcg.edu.br (A.G.B.d.L.); douglasasl@ufcg.edu.br (D.A.S.L.); 2 Department of Agricultural Engineering, Federal University of Campina Grande, Campina Grande 58429-900, Brazil; rossanamff@gmail.com (R.M.F.d.F.); alexandrejmq@gmail.com (A.J.d.M.Q.); francislainesuelis@gmail.com (F.S.d.S.); lumaratatielyea@gmail.com (L.T.S.A.); tthallisma@gmail.com (T.L.B.d.L.); wiltonps@uol.com.br (W.P.d.S.); valentim_henrique@hotmail.com (H.V.M.); eugenia_telys@hotmail.com (E.T.d.V.S.); caciana.cavalcanti@professor.ufcg.edu.br (C.C.C.); josivanda@gmail.com (J.P.G.); 3 Unit of Food Technology Academic, Federal University of Campina Grande, Pombal 58840-000, Brazil; pluvia.oliveira@professor.ufcg.edu.br

**Keywords:** *Malpighia emarginata*, *Psidium guajava*, *Eugenia uniflora*, Hausner factor, Carr index

## Abstract

Guava, pitanga and acerola are known for their vitamin content and high levels of bioactive compounds. Thus, the preparation of combinations of these fruits comprises a blend with high nutraceutical potential, yielding a strong and attractive pigmentation material. In this study, the influence of different proportions of maltodextrin on the lyophilization of a blend of guava, acerola and pitanga was evaluated considering not only the physicochemical, physical and colorimetric parameters but also the bioactive compounds in the obtained powders. The blend was formulated from the mixture and homogenization of the three pulps in a ratio of 1:1:1 (m/m), then maltodextrin was added to the blend, resulting in four formulations: blend without adjuvant (BL0), and the others containing 10% (BL10), 20% (BL20) and 30% (BL30) maltodextrin. The formulations were lyophilized and disintegrated to obtain powders. The powders were characterized in terms of water content, water activity, pH, total titratable acidity, ash, total and reducing sugars, ascorbic acid, total phenolic content, flavonoids, anthocyanins, carotenoids, lycopene, color parameters, Hausner factor, Carr index, angle of repose, solubility, wettability and porosity. All evaluated powders showed high levels of bioactive compounds and the increase in maltodextrin concentration promoted positive effects, such as reductions in water content, water activity and porosity and improved flow, cohesiveness and solubility characteristics.

## 1. Introduction

Red fruits are commonly linked to certain temperate climate species; nevertheless, the term also aptly applies to a variety of tropical fruits that share reddish pigments, indicating the presence of bioactive antioxidant compounds. Among these examples are guava, acerola and pitanga, which belong to different genres but share these common characteristics.

Guava (*Psidium guajava* L.) is appreciated by consumers, and it is rich in dietary fiber and bioactive compounds with antioxidant activity [1], possessing the ability to prevent the incidence of chronic and degenerative diseases [2], including arthritis, arteriosclerosis and cancer [3]. The pitanga (*Eugenia uniflora* L.) has a high nutritional value, standing out for the amount of vitamins, polyphenols and antioxidants; in addition, it has an exotic flavor and aroma [4] and also several biological activities, such as anti-inflammatory, antidiabetic, antimalarial and diuretic. Acerola (*Malpighia emarginata* L.) is rich in vitamin C, amino acids and phenolic compounds, mainly flavonoids, anthocyanins and carotenoids. It is widely disseminated in several countries [5,6] and has several biological functions, such as antihyperglycemic effect, anticancer activity against lung cancer and protective effect against genotoxicity [7].

Despite the sensory and nutritional advantages of consuming fresh fruit, its commercialization in this state involves difficulties and significant losses due to its short shelf life. In addition, consumers often find a lack of practicality, as their demand is met by derivatives obtained from processing, leading to the consumption of more juices, jellies and other products.

Each fruit has its own bioactive compounds, such as polyphenols and carotenoids, which have antioxidant properties and may contribute to health. The combination of fruits (acerola, guava and pitanga) in the form of a blend can result in a higher concentration of such compounds, enhancing the benefits for the organism and also providing unique gustatory and aromatic experiences.

Under ideal processing conditions, it is possible to generate dehydrated products with safe water content levels, making them healthy, easy to prepare and consume on a daily basis, with characteristics close to those of fresh products [8]. Among the drying methods, lyophilization is the process used by the food industry to produce the highest quality dry products [9], more effectively promoting the preservation of heat-sensitive nutrients and providing stability of these compounds, since it operates at low temperatures and under high vacuum [10].

It is difficult to produce certain freeze-dried products because they have a low glass transition temperature, requiring the addition of an additive to aid the drying process [9]. Several additives are used; however, maltodextrin is one of the most commonly used, especially to obtain fruit powders, having characteristics such as high solubility in water, the ability to generate solutions with low viscosity, being tasteless, easily biodegradable and providing good cost–benefit value for money [11,12]. In addition, it supports the shelf life of the product by providing a protective barrier against moisture absorption and helps to improve the texture and mouthfeel of the reconstituted product.

It is important to note that the effects of adding maltodextrin may vary depending on the proportion used, the specific characteristics of the raw materials, the freeze-drying process and other processing conditions [13]. Therefore, it is essential to carry out specific studies and tests to optimize the formulation according to the desired objectives, ensuring the preservation of bioactive compounds and obtaining the characteristics met for the final product. Thus, the objective was to evaluate the influence of different proportions of maltodextrin in the formulation of a blend of freeze-dried tropical red fruits (guava, pitanga and acerola), characterizing them in terms of physicochemical, physical, colorimetric parameters and bioactive compounds.

## 2. Results and Discussion

### 2.1. Physicochemical Characterization of Red Fruit Blend Powders

Table 1 presents the average values with the respective standard deviations of the physicochemical parameters evaluated in the blend powders obtained by lyophilization.

It was observed that the water content of all powdered samples differed significantly from each other, with a reduction as the maltodextrin concentration was high. A similar trend occurred in relation to water activity (a_w_), demonstrating that the added amount of maltodextrin helped in the lyophilization process and reduced the water content and a_w_. Similar behavior was reported by Barroso et al. [14] in lyophilized mangaba pulp (*Hancornia speciosa* Gomes) with the addition of maltodextrin from 0 to 30%, resulting in water contents ranging from 11.44 to 1.16 g/100 g wb and by Andrade et al. [15] in lyophilized guava pulp with 14, 21 and 28% maltodextrin, obtaining a_w_ of 0.150, 0.103 and 0.073, respectively.

Rahman [16] stated that products with a_w_ < 0.6 have low availability of water to be used in biochemical reactions, which promotes prolonged stability, as long as they are packaged in a way that there is no absorption of water from the environment. This a_w_ limit reported by the author is in line with the values obtained in the present study in all powders.

Costa et al. [17] reported a water content of 4.37 g/100 g wb in lyophilized Palmer mango pulp with 20% maltodextrin, which is lower than the powders in the present study. Similar values were quantified by Almeida et al. [18], evaluating lyophilized jabuticaba peels without any adjuvants, with an a_w_ of 0.320.

The pH of the powders is characteristic of highly acidic products (pH < 4.0), resulting from the fruits used to prepare the blend. Some additives can chemically react when combined, which can also affect the pH; but in the case of maltodextrin incorporation, there was no effect on this parameter. The samples did not differ significantly, except between BL0 (3.71) and BL10 (3.64), which still had close values. The powdered mixture of acerola and lyophilized pineapple without adjuvants was also classified as highly acidic, with a pH of 3.52 [19].

It was observed that the BL0 powder (control) had the highest total titratable acidity, with a significant reduction in the samples with the addition of maltodextrin in relation to the control; however, it is noted that when exceeding the concentration of 20% of maltodextrin, there was no significant increase in acidity, with similar BL20 and BL30 values. The high acidity in the red fruit blend powders is due to the reduction in the water content that concentrates the organic acids present in the samples, as evidenced by Barroso et al. [14]. The tendency of acidity reduction with the increase in maltodextrin concentration was also verified by Andrade et al. [15] in powders of lyophilized guava pulp with maltodextrin (14 to 28%), ranging from 1.72 to 1.09 g citric acid/100 g db.

High ash contents were quantified in the powders and the incorporation of the adjuvant significantly reduced the mineral content, corroborating what was verified by Ermis, Guner and Yilmazz [20] in lyophilized hazelnut milk with 0, 5, 10 and 15% maltodextrin, obtaining values from 2.12 to 1.16 g/100 g db.

As the concentration of maltodextrin increased, the total sugar (TS) content of the powders increased and there was a reduction in the reducing sugar content. As a result of maltodextrin being a carbohydrate that contains a large amount of glucose units in its structure, when it is added to the fruit blend, the total amount of carbohydrates present in the product increases. However, freeze-drying can result in a decrease in the content of reducing sugars in the final product, since the process involves the removal of water by sublimation, which can lead to the formation of reaction products involving the sugars present. Maciel et al. [21] also observed similar behavior for total sugars in lyophilized *cupuaçu* pulp powders with 5, 15 and 25% maltodextrin, with contents of 47.72, 55.62 and 62.32 g/100 g db, respectively.

### 2.2. Bioactive Compounds

The levels of bioactive compounds in the powders of the lyophilized formulations are presented in Table 2. It was verified that the increase in the proportion of maltodextrin resulted in reductions in all parameters, which were gradually reduced with the percentage increases of incorporation, with statistically significant effects. Part of the reduction is the effect of incorporating the material into the samples itself, considering that maltodextrin is free of bioactive compounds. But it is also possible that the encapsulation promoted by the additive affected the extraction of these compounds and the consequent measurement.

Although the addition of maltodextrin reduced the contents of most bioactive compounds, they still remain at considerably high levels. For example, in the BL30 sample, ascorbic acid still exceeds the daily intake recommended by legislation [22], which is 45 mg for adults, with a much higher amount in 100 g. The results obtained were higher than those reported for lyophilized Palmer mango pulp with 20% maltodextrin (90.46 mg/100 g db evaluated by Costa et al. [17] and for lyophilized *cubiu* (*Solanun sessiflorum* Dunal) pulp (11.86 mg/100 g) as analyzed by Oliveira, Silva e Silva [23].

In total phenolic content (TPC), the greatest decline (82.5%) was observed in the BL30 sample, with the highest concentration of adjuvant, in relation to the control sample (BL0). Identical behavior was found in the phenolic extract of wild pomegranate peel, in which the content of phenolic compounds increased from 96.39 mg GAE/100 g (db) in a 1:1 ratio (extract: maltodextrin) to 49.33 mg GAE/100 g (db) when the additive amount was increased to 1:10 [24]. However, even the BL30 sample presents values higher than those determined in *guabijú* pulp (*Myrcianthes pungens*) lyophilized without adjuvants, studied by Detoni et al. [25], which presented levels of 2003.39 and 2179.95 mg GAE/100 g db; and values similar to those found in mixed pulp powders of jambolan and acerola produced by drying in a foam layer (50–80 °C) with different additives were observed, with values ranging from 5144.35 to 6999.34 1 mg/100 g db [26].

According to Souza et al. [27], all samples evaluated in the present study can be classified as having a high content of total phenolic content (above 500 mg GAE/100 g). The presence of phenolic compounds in food products is of great importance, as they are natural bioactive molecules that demonstrate antioxidant, antimicrobial, anti-inflammatory and antiproliferative activities, among others [28].

For flavonoids and anthocyanins, the reductions in the BL30 samples in relation to the BL0 control were 59 and 45.89%, respectively. Although there was a considerable reduction, the blend with 30% of the additive still showed a flavonoid content close to that determined in the grape foam powder (BRS Rúbea×IAC 1398-21) lyophilized with only half the addition of maltodextrin (15%), which was reported by the authors as 11.01 mg/100 g [29], indicating the richness in flavonoids of the powders studied in the present work.

Much higher values of anthocyanins were reported for lyophilized myrtle pulp powder with 20% maltodextrin, with levels from 145.56 to 146.12 mg/100 g db before storage [30]. However, despite the reduced content of anthocyanins in the red fruit blend powders, these pigments are derived from flavonols and are responsible for the variation in the red hue of various fruits with acidic pH, with important bioactivities beneficial to health and are widely used as natural dyes [28].

Among the determined compounds, carotenoids showed the second-largest decline (79.63%) between BL0 and BL30, while lycopene showed a maximum reduction of 68.31%. Even with the reductions, the red fruit blend powders still had higher carotenoid contents than the lyophilized *cubiu* (*Solanun sensiflorum* Dunal) pulp without adjuvants, which had 0.246 mg/100 g [23].

### 2.3. Color Measurement

**Figure 1:**
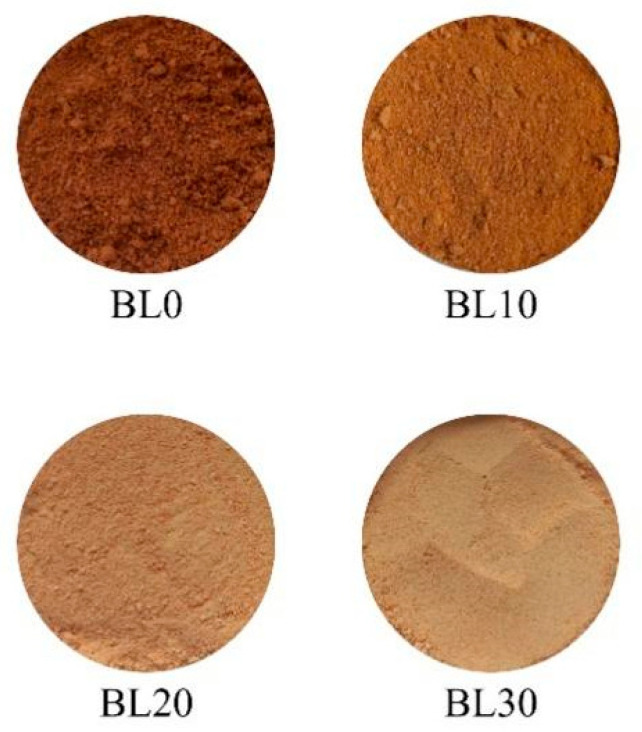
Images of the lyophilized tropical red fruit blend powders with the addition of maltodextrin (concentration of maltodextrin in the blend: BL0 (0%); BL10 (10%); BL20 (20%); and BL30 (30%)).

The averages and standard deviations referring to the colorimetric parameters of the tropical red fruit blend powders with the addition of maltodextrin obtained through lyophilization are presented in Table 3 and Figure 1, with their images.

All samples showed brightness (L*) lower than 50, on a scale from 0 (black) to 100 (white), with samples BL0 and BL10 being considered the darkest samples, BL20 and BL30 the lightest, according to can be seen in Figure 1. The addition of maltodextrin provided a statistical increase in this parameter, indicating the transition to lighter tones, a result of its characteristic brightness, greater than that of the studied pulp samples. The increase in the L* parameter is due to the use of maltodextrin, which tends to whiten the sample, in addition to diluting the pigments in the sample, thereby making it appear lighter [21].

The positive a* values are a consequence of the predominance of the red hue in relation to the green one, observing that the increase in the proportion of the additive led to reductions. The intensity of yellow (+b*), the predominant hue in BL0, was also progressively reduced with the addition of maltodextrin. In their study of a blend composed of freeze-dried acerola and pineapple, Silva et al. [19] found L* of 42.85 and red (+a*) and yellow (+b*) intensities of 36.39 and 25.75, respectively. In their study characterizing flours obtained from fruit residues, Menezes Filho and Castro [31] found L* of 58.67, +a* of 12.54 and +b* of 27.60 for the flour obtained from the rind and pulp of ripe guava.

The overall effect of additive addition on the a* and b* chromaticity components is given by the chroma and hue angle. The chroma (C*) values were also statistically reduced with the increase in the proportion of maltodextrin. When these values are close to zero, they correspond to neutral colors (gray tones), while values close to 60 indicate bright colors [32]. Considering the values found, it appears that the powders tended to shift towards neutral colors, more grayish, with increases in the proportion of additives.

In the hue angle (°), there was a predominance of the yellow hue in the BL0 sample (h* > 45°), transitioning to red with the increase in the percentage of maltodextrin. The hue angle assumes values in the intervals of 0° for red, 90° for yellow, 180° for green and 270° for blue [33].

### 2.4. Physical Characterization

Table 4 presents the mean results and standard deviations of the physical parameters of the powders of the lyophilized formulations with different proportions of maltodextrin.

The Hausner factor (HF) and the Carr index (CI) are used in the evaluation of the properties of cohesion and flow of powders, with the HF being related to the friction between the particles, while IC indicates their aggregation capacity [34]. Powders that present HF lower than 1.2 are classified as low cohesiveness, between 1.2 and 1.4 have intermediate cohesiveness and HF > 1.4 are considered high cohesiveness. For flow index, powders with CI values between 15 and 20% have good flow, between 20 and 35% have poor flow, between 35 and 45% also have poor flow, and >45 have very poor flow [35].

The addition of maltodextrin to the blend caused a decrease in HF and CI, with the cohesion factor (HF) being in the intermediate cohesiveness range (between 1.22 and 1.37), except for BL30, which showed low cohesiveness. According to the Carr index (CI), the blend powders showed good fluidity (BL20 and BL30) and poor fluidity (BL0 and BL10). In their study on the effects of adding carrier agents (maltodextrin, gum Arabic and dextrin) to lyophilized red dragon fruit (*Hylocereus polyrhizus*) pulp powders, Alves et al. [36] observed behavior similar to that of the present study. They noted HF values ranging from 1.29 and 1.75 and CI from 22.61 to 43.00%. In their study on the effects of gum Arabic and inulin additives on the lyophilization of *Hibiscus acetosella* extract, Mar et al. [37] reported a Hausner factor between 1.3 and 1.4.

The angle of repose was smaller in the sample that did not receive maltodextrin, differing significantly from the other formulations, which, in turn, did not present statistical differences between them. Rocha et al. [38] observed the same behavior analyzing the flow of mango pulp powders containing different concentrations of maltodextrin. These results can be attributed not only to the composition of the samples but also to the way the material was pulverized. The samples were ground after dehydration, in a way that the sample without additive could have generated particles with less rough surfaces. The angle of repose is used to characterize the flow properties of solids, relating to interparticle friction or resistance to motion between particles. In general, the smaller the angle of repose, the better the fluidity of the powder. Geldart et al. [39] established a limit in which powders with angles of repose > 50° present flow difficulties and those with values < 30° flow with good fluidity.

The addition of maltodextrin significantly affected the solubility of the blend powders, which increased significantly in direct relation to the additive concentration. This fact is associated with the high solubility of the carrier agent used and with the particle size of the material produced (Figure 1), since the smaller the particle size, the greater the surface area available for hydration. Another aspect that must be considered is the dispersion capacity of the particles, given that less agglomeration favors solubility [40].

In their study on the effects of carrier agents at different concentrations on the physicochemical properties of lyophilized date powder, Seerangurayar et al. [41] reported a similar trend, in which the control powder showed significantly lower solubility compared to the powders added with maltodextrin (DE 10), whose solubility values ranged from 80 to 81%. Ribeiro et al. [42] found a higher value than the one found in the present work, i.e., 94%, for the solubility of lyophilized acerola pulp powder with 19% maltodextrin (DE 20). In addition to the high solubility of maltodextrin, the dehydration method applied can generate a more porous product with greater rehydration capacity.

The wettability was higher in the sample without additive, decreasing with the increase in the percentage of maltodextrin incorporation. Similar behavior was described by Andrade et al. [15] for the pulp of guava (*Eugenia stipitata*) lyophilized with different concentrations of maltodextrin (14, 21 and 28%). The reduction in the wettability of the samples with maltodextrin must be attributed to a lower wettability of the additive itself in relation to the blend, resulting in longer immersion times with the increase in the added percentages of the additive. The fact that the BL0 sample does not have an additive in its formulation, generates a higher water content in the product which, in turn, favors the increase in the size of the particles. According to Custodio et al. [43], a larger particle size results in a shorter wetting time.

The porosity of the powders with the addition of maltodextrin was reduced in relation to the control, reaching the lowest value in the BL30 sample. The reduction of porosity in stored powders is important for the preservation of their characteristics, since powders with high porosity have many empty spaces, allowing for a greater presence of oxygen, which can trigger oxidation reactions [44]. Thus, maltodextrin has a potentially protective effect during storage.

## 3. Materials and Methods

### 3.1. Raw Materials and Processing

Acerola (*Malpighia emarginata*), guava (*Psidium guajava*) and pitanga (*Eugenia uniflora* L.) fruits were collected between January and March 2020, in the municipalities of Petrolina (latitude 9°23′39″ S, longitude 40°30′35″ W, altitude 380 m) and Bonito (latitude 8°28′13″ S, longitude 35°43′35″ W, altitude 423 m), both located in the state of Pernambuco, Brazil.

The fruits were selected at the maturation stage when the acerola and pitanga had completely red skin, and the guava had completely yellow skin, with no visible injuries. They were then washed in running water to eliminate foreign materials and sanitized by immersion in chlorinated water (50 ppm) for 15 min, followed by rinsing in potable water to remove excess sanitizer. Then they were pulped in a horizontal pulper (Laboremus^®^, model DF-200, Campina Grande, Paraíba, Brazil). The three whole pulps obtained (acerola, guava and pitanga) were used to prepare the blend of red fruits, using a ratio of 1:1:1 (g/g) for the formulation (this proportion was determined through preliminary tests), with the pulps weighed individually and homogenized during 2 min in a domestic blender (2000 rpm) (Arno^®^, Power Mix model, Itapevi, São Paulo, Brazil).

### 3.2. Preparation of Formulations and Lyophilization

For the lyophilization process, four blend formulations were prepared, one control, without the addition of maltodextrin (BL0), and three containing maltodextrin (MOR-REX^®,^ São Paulo, Brazil) with a dextrose equivalent (DE) of 10 at concentrations of 10% (BL10), 20% (BL20) and 30% (BL30), being homogenized in a domestic blender (Arno^®^, Power Mix model, Itapevi, São Paulo, Brazil) for 3 min.

Initially, the four formulations with the blend were placed in plastic trays for ice (22.9 × 14.6 × 3.8 cm) and subjected to freezing in a freezer (−18 °C) for 48 h. After this step, the materials were taken to the lyophilizer (Liobras^®^, model BL101, São Carlos, São Paulo, Brazil) and kept in the equipment at −50 °C (<500 µHg) for 72 h.

After the lyophilization of the samples, they were disintegrated with the aid of a mortar and pestle, and then the physicochemical, physical, bioactive compounds and color analyses were performed.

### 3.3. Characterization of Lyophilized Blend Formulation Powders

#### 3.3.1. Physicochemical Characterization

The physicochemical analyses of the blend powders were performed in quadruplicate using the methodologies proposed by the AOAC [45]: water content, performed by the standard oven method (QUIMIS^®^, model Q319V, Diadema, São Paulo, Brazil) at 105 ± 3 °C, up to constant mass; total titratable acidity determined by the acidimetric method, using 0.1 M sodium hydroxide solution, with the results expressed as a percentage of citric acid; ash determined by calcining the sample in a muffle at 550 ± 5 °C; the TSS/ATT ratio was estimated by the quotient of the values of total soluble solids and total titratable acidity.

Water activity was determined by direct reading of the sample in a dew point hygrometer (Aqualab, model 3TE, Decagon, Washington, DC, USA) at a temperature of 25 °C.

The pH was determined by the potentiometric method [45], with a pH meter (Tecnal, model TEC-2, São Paulo, São Paulo, Brazil), previously calibrated with buffer solutions of pH 4.0 and 7.0.

The total soluble sugar contents (g/100 g) were determined using the methodology of Yemm and Willis [46] and for the reducing sugar contents (g/100 g) the methodology of Miller [47] was used. Both analyses were performed using a spectrophotometer (Coleman^®^, model 35 D, Santo André, São Paulo, Brazil).

#### 3.3.2. Bioactive Compounds

The ascorbic acid content (mg ascorbic acid/100 g) was determined based on the protocol by Oliveira, Godoy and Prado [48]; the content of total phenolic content (TPC) was quantified by the method described by Waterhouse [49]; total flavonoids (mg/100 g) and total anthocyanins (mg/100 g) by the methodology described by Francis [50]; total carotenoids (g/100 g) according to Lichtenthaler [51]; lycopene according to Nagata and Yamashita [52]. All absorbance readings during the analyses were performed in a spectrophotometer (Coleman^®^, model 35 D, Santo André, São Paulo, Brazil).

#### 3.3.3. Color Measurement

Colorimetric parameters were evaluated using a portable spectrophotometer (MiniScan, Hunter Lab XE Plus, model 4500 L, Hunter Associates Laboratory, Reston, VA, USA). Color coordinate readings were performed using the CIELAB system: L* (brightness), a* (transition from green to red) and b* (transition from blue to yellow); hue angle (h*) and color saturation or chroma (C*) were calculated according to Equations (1) and (2), respectively.

(1) $$h{}^{*}\;=\tan^{-1}(b^{*}/a^{*})$$

(2) $$C^{*}=\sqrt{{(a{}^{*}}^{2}+{b{}^{*}}^{2})}$$

#### 3.3.4. Physical Characterization

Solubility was determined according to the method described by Cano-Chauca et al. [53].

The wettability was determined using the Schubert method [54], expressed by the ratio between the mass (g) and the time required for the sample to disappear from the surface (min).

The Carr index (CI) and the Hausner factor (HF) were determined using the methodology of Santhalakshmy et al. [35], calculated from apparent density (ρ_ap_) and compacted density (ρ_c_) data, according to Equations (3) and (4):

(3) $$\mathrm{CI}=\frac{\rho_{c}-\rho_{\mathrm{ap}}}{\rho_{\mathrm{ap}}}100$$

(4) $$HF=\frac{\rho_{c}}{\rho_{ap}}$$

For the evaluation of the angle of repose, the methodology described by Aulton [55] was used. The porosity was calculated through the expression whose relation relates to the apparent density and the absolute density.

The apparent density was determined from the relationship between the mass and the volume occupied in the cylinder, according to Goula and Adamopoulos [56]. The results are expressed in g/cm^3^. To determine the compacted density, the method described by Tonon [57] was used. The absolute density was determined using a pycnometer, where the powder sample and hexane were added as immiscible liquids, at a temperature of 25 °C. With the obtained data, the relationship between the sample mass and the volume of the pycnometer was calculated.

### 3.4. Statistical Analysis

All analyses were performed in quadruplicate and the data obtained were statistically evaluated using the Assistat^®^ software version 7.7, with the experiments carried out in a completely randomized design (CRD) and with the comparison between means by Tukey’s test at a significance level of 5% [58].

## 4. Conclusions

All powders obtained from the evaluation of the tropical red fruit blend have high levels of bioactive compounds. The increase in maltodextrin concentration during the lyophilization of the tropical red fruit blend promotes positive effects on the powders, such as reductions in water content, water activity and porosity, providing better flow, cohesiveness and solubility characteristics. Among the evaluated formulations, BL20 and BL30 with the highest concentrations of maltodextrin are likely to have better stability during storage, with better flow characteristics.

## Figures and Tables

**Table 1:** Mean values and standard deviations of the physicochemical parameters of the lyophilized tropical red fruit blend powders with the addition of maltodextrin.

Parameters	BL0	BL10	BL20	BL30
Water content ^1^	18.11 ± 0.46 a	8.67 ± 0.23 b	6.23 ± 0.18 c	5.42 ± 0.20 d
Water activity (a_w_)	0.366 ± 0.04 a	0.354 ± 0.03 b	0.246 ± 0.01 c	0.243 ± 0.02 c
pH	3.71 ± 0.04 a	3.64 ± 0.03 b	3.67 ± 0.02 ab	3.69 ± 0.01 ab
Total titratable acidity ^2^	14.36 ± 0.11 a	7.99 ± 0.11 b	7.32 ± 0.07 c	7.15 ± 0.11 c
Ashes ^3^	3.80 ± 0.10 a	2.04 ± 0.06 b	1.34 ± 0.02 c	0.98 ± 0.01 d
Total sugars ^3^	18.89 ± 0.08 d	20.51 ± 0.92 c	21.57 ± 0.15 b	24.84 ± 0.24 a
Reducing sugars ^3^	4.85 ± 0.09 a	2.91 ± 0.04 b	2.24 ± 0.08 c	1.89 ± 0.07 d

Concentration of maltodextrin in the blend: BL0 (0%); BL10 (10%); BL20 (20%); and BL30 (30%). ^1^ (g/100 g wb); ^2^ (g citric acid/100 g db); ^3^ (g/100 g db). Means followed by the same lowercase letters on the lines do not differ statistically by Tukey’s test at 5% probability (*p* < 0.05).

**Table 2:** Mean values and standard deviations of bioactive compounds in lyophilized red fruit blend powders with added maltodextrin.

Parameters	BL0	BL10	BL20	BL30
Ascorbic acid ^1^	15,563.92 ± 4.65 a	10,721.15 ± 2.70 b	8993.97 ± 3.06 c	6084.99 ± 2.03 d
Total phenolic content ^2^	18,919.49 ± 4.06 a	10,399.14 ± 4.93 b	6448.62 ± 1.56 c	3311.05 ± 5.18 d
Flavonoids ^1^	25.83 ± 0.12 a	21.77 ± 0.36 b	12.22 ± 0.25 c	10.59 ± 0.20 d
Anthocyanins ^1^	15.32 ± 0.28 a	11.86 ± 0.28 b	10.33 ± 0.27 c	8.29 ± 0.15 d
Carotenoids ^1^	108.92 ± 0.76 a	69.09 ± 0.76 b	33.86 ± 0.89 c	22.19 ± 0.76 d
Lycopene ^1^	0.508 ± 0.005 a	0.363 ± 0.004 b	0.228 ± 0.005 c	0.161 ± 0.008 d

Concentration of maltodextrin in the blend: BL0 (0%); BL10 (10%); BL20 (20%); and BL30 (30%). ^1^ mg/100 g db; ^2^ mg GAE*/100 g db; * Equivalent to gallic acid. Means followed by the same lowercase letters on the lines do not differ statistically by Tukey’s test at 5% probability (*p* < 0.05).

**Table 3:** Mean values and standard deviations of the colorimetric parameters of lyophilized tropical red fruit blend powders with added maltodextrin.

Parameters	BL0	BL10	BL20	BL30
Brightness (L*)	14.60 ± 0.56 d	25.13 ± 0.14 c	33.85 ± 0.31 b	41.84 ± 0.13 a
Intensity of red (+a*)	16.39 ± 0.23 a	16.17 ± 0.04 a	11.83 ± 0.08 b	11.59 ± 0.13 b
Intensity of yellow (+b*)	23.30 ± 0.08 a	14.67 ± 0.31 b	12.85 ± 0.16 c	9.86 ± 0.33 d
Chroma (C*)	28.49 ± 0.30 a	21.83 ± 0.09 b	17.47 ± 0.15 c	15.21 ± 0.25 d
Hue angle (h*) (^o^)	54.88 ± 0.62 a	42.22 ± 0.06 c	47.37 ± 0.36 b	40.39 ± 0.95 d

Concentration of maltodextrin in the blend: BL0 (0%); BL10 (10%); BL20 (20%); and BL30 (30%). Means followed by the same lowercase letters on the lines do not differ statistically by Tukey’s test at 5% probability (*p* < 0.05).

**Table 4:** Mean values and standard deviations of physical parameters of lyophilized tropical red fruit blend powders with added maltodextrin.

Parameters	BL0	BL10	BL20	BL30
Hausner factor	1.37 ± 0.00 a	1.25 ± 0.01 b	1.22 ± 0.01 c	1.18 ± 0.01 d
Carr index ^1^	27.00 ± 0.00 a	20.25 ± 0.50 b	18.25 ± 0.50 c	15.50 ± 1.00 d
Angle of repose ^2^	26.57 ± 0.00 b	29.75 ± 0.00 a	29.76 ± 0.00 a	29.83 ± 0.01 a
Solubility ^1^	49.62 ± 0.29 d	74.10 ± 1.01 c	90.17 ± 1.18 b	96.05 ± 1.48 a
Wettability ^3^	2.77 ± 0.13 a	0.76 ± 0.01 b	0.71 ± 0.001 b	0.66 ± 0.01 c
Porosity ^1^	83.99 ± 0.09 a	66.36 ± 0.57 b	66.07 ± 0.31 b	61.94 ± 0.46 c

Concentration of maltodextrin in the blend: BL0 (0%); BL10 (10%); BL20 (20%); and BL30 (30%). 1 (%); 2 (°); 3 (g/min). Means followed by the same lowercase letters on the lines do not differ statistically by Tukey’s test at 5% probability (*p* < 0.05).

## Data Availability

Data can be digitized from the graphs or requested from the corresponding author.
